# Proper understanding of recurrent stress urinary incontinence treatment in women (PURSUIT): a randomised controlled trial of endoscopic and surgical treatment

**DOI:** 10.1186/s13063-022-06546-9

**Published:** 2022-08-03

**Authors:** L. Clark, B. Fitzgerald, S. Noble, S. MacNeill, S. Paramasivan, N. Cotterill, H. Hashim, S. Jha, P. Toozs-Hobson, T. Greenwell, N. Thiruchelvam, W. Agur, A. White, V. Garner, M. Cobos-Arrivabene, C. Clement, M. Cochrane, Y. Liu, A. L. Lewis, J. Taylor, J. A. Lane, M. J. Drake, C. Pope

**Affiliations:** 1 Department of Population Health Sciences, Bristol Medical School, University of Bristol, Bristol, UK; 2 Bristol Urological Institute, Southmead Hospital, North Bristol NHS Trust, Bristol, UK; 3 Department of Urogynaecology, Sheffield Teaching Hospitals NHS Foundation Trust, Jessop Wing, Tree Root Walk, Sheffield, UK; 4 Department of Urogynaecology, Birmingham Women’s & Children’s Hospital NHS Foundation Trust, Birmingham, UK; 5 Department of Urology, University College London Hospital, London, UK; 6 Department of Urology, Cambridge University Hospitals NHS Trust, Cambridge, UK; 7 Department of Obstetrics and Gynaecology, NHS Ayrshire and Arran, University Hospital Crosshouse, Kilmarnock, UK; 8 Patient and Public Involvement (PPI) Representative, Bristol, UK; 9 Bristol Trials Centre (BTC), University of Bristol, Bristol, UK; 10 Department of Translational Health Sciences, Bristol Medical School, University of Bristol, Bristol, UK

**Keywords:** PURSUIT, Recurrent stress urinary incontinence, Endoscopic bulking injections, Surgery, Colposuspension, Autologous fascial sling, Artificial urinary sphincter, Randomised controlled trial, International Consultation on Incontinence Questionnaire – Urinary Incontinence – Short Form (ICIQ-UI-SF), Qualitative

## Abstract

**Background:**

Women with stress urinary incontinence (SUI) experience urine leakage with physical activity. Currently, the interventional treatments for SUI are surgical, or endoscopic bulking injection(s). However, these procedures are not always successful, and symptoms can persist or come back after treatment, categorised as recurrent SUI. There are longstanding symptoms and distress associated with a failed primary treatment, and currently, there is no consensus on how best to treat women with recurrent, or persistent, SUI.

**Methods:**

A two-arm trial, set in at least 20 National Health Service (NHS) urology and urogynaecology referral units in the UK, randomising 250 adult women with recurrent or persistent SUI 1:1 to receive either an endoscopic intervention (endoscopic bulking injections) or a standard NHS surgical intervention, currently colposuspension, autologous fascial sling or artificial urinary sphincter. The aim of the trial is to determine whether surgical treatment is superior to endoscopic bulking injections in terms of symptom severity at 1 year after randomisation. This primary outcome will be measured using the patient-reported International Consultation on Incontinence Questionnaire - Urinary Incontinence - Short Form (ICIQ-UI-SF). Secondary outcomes include assessment of longer-term clinical impact, improvement of symptoms, safety, operative assessments, sexual function, cost-effectiveness and an evaluation of patients’ and clinicians’ views and experiences of the interventions.

**Discussion:**

There is a lack of high-quality, randomised, scientific evidence for which treatment is best for women presenting with recurrent SUI. The PURSUIT study will benefit healthcare professionals and patients and provide robust evidence to guide further treatment and improve symptoms and quality of life for women with this condition**.**

**Trial registration:**

International Standard Randomised Controlled Trials Number (ISRCTN) registry ISRCTN12201059. Registered on 09 January 2020

## Administrative information

Note: the numbers in curly brackets in this protocol refer to SPIRIT checklist item numbers. The order of the items has been modified to group similar items (see http://www.equator-network.org/reporting-guidelines/spirit-2013-statement-defining-standard-protocol-items-for-clinical-trials/).

**Table:** 

Title {1}	Proper Understanding of Recurrent Stress Urinary Incontinence Treatment in women (PURSUIT): A Randomised Controlled Trial of Endoscopic and Surgical Treatment.
Trial registration {2a and 2b}.	ISRCTN registry, ID: ISRCTN12201059. Registered on 09 January 2020.
Protocol version {3}	Version 4.0, 03 September 2020
Funding {4}	This research is funded by the National Institute for Health and Care Research (NIHR) Health Technology Assessment (HTA) programme (project number 17/95/03).
Author details {5a}	^1^ Department of Population Health Sciences, Bristol Medical School, University of Bristol, Bristol, UK. ^2^ Bristol Urological Institute, Southmead Hospital, North Bristol NHS Trust, Bristol, UK. ^3^ Department of Urogynaecology, Sheffield Teaching Hospitals NHS Foundation Trust, Jessop Wing, Tree Root Walk, Sheffield, UK. ^4^ Department of Urogynaecology, Birmingham Women’s & Children's Hospital NHS Foundation Trust, Birmingham, UK. ^5^ Department of Urology, University College London Hospital, London, UK. ^6^ Department of Urology, Cambridge University Hospitals NHS Trust, Cambridge, UK. ^7^ Department of Obstetrics and Gynaecology, NHS Ayrshire and Arran, University Hospital Crosshouse, Kilmarnock, UK. ^8^ Patient and Public Involvement (PPI) Representative, Bristol, UK. ^9^ Bristol Trials Centre (BTC), University of Bristol, Bristol, UK. ^10^ Department of Translational Health Sciences, Bristol Medical School, University of Bristol, Bristol, UK. ^*^Corresponding author
Name and contact information for the trial sponsor {5b}	This trial is sponsored by Research and Innovation, North Bristol NHS Trust, Floor 3 Learning and Research Building, Southmead Hospital, Westbury-on-Trym, Bristol, BS10 5NB. Telephone: 0117 414 9330. Email: researchsponsor@nbt.nhs.uk.
Role of sponsor {5c}	The PURSUIT study was designed in response to the HTA funding call 17/95 - Treatments for women with recurrent stress urinary incontinence after failed primary surgery. North Bristol NHS Trust was involved with the design of the study, preparation and approval of the study protocol and signing off the validation of the study database for data collection. The sponsor will ensure interim and final analysis of the data is conducted and will review and contribute to manuscripts for publication.

## Introduction

### Background and rationale {6a}

Urinary leakage with physical effort or exertion, such as coughing, sneezing or exercising, is known as stress urinary incontinence (SUI), and primary SUI affects a quarter (16–35%) of women after pregnancy. Until recently, the most common surgical treatment for primary SUI was a midurethral tape (MUT), an operation which helps to support the bladder exit (urethra). Alternative surgical options include colposuspension, autologous fascial sling or artificial urinary sphincter (AUS). Endoscopic urethral bulking injections are a minimally invasive surgical alternative. In many cases symptoms can persist or come back, known as persistent or recurrent SUI. The treatment options generally used are the same as those for primary SUI.

Little is known about the chance of cure or potential treatment-related problems for those women with ongoing SUI after treatment. There is also no consensus on how to treat women who have had failed primary continence surgery. A study by Tincello et al. surveyed patients and clinicians about this question [1]. “No consensus on what is the correct treatment” was achieved by a clinician survey and patient views were highly individual. There is a problematic lack of high-quality evidence for the best treatment for recurrent SUI [2, 3].

The underlying mechanism of SUI may be either:

1. *Urethral hypermobility*, where the sphincter muscle is fundamentally normal, but is prevented from functioning due to impairment of its ligamentous support, or
2. *Intrinsic sphincter deficiency*, where the sphincter muscle is not normal, because the nerves to the muscle, or the muscle itself, are damaged. Consequently, there is weakened resistance by the sphincter.

The National Institute for Health and Care Excellence (NICE) guidelines NG123 on Urinary Incontinence in Women [4] suggests that women whose primary surgical procedure for SUI has failed (including women whose symptoms have returned) should be referred to tertiary care for assessment (such as repeat urodynamic testing, including additional tests such as imaging and urethral function studies), and discussion of treatment options by the multidisciplinary team.

In primary SUI, NICE recommends pelvic floor muscle training and, if this fails, surgery is an option. Surgical failure rates after MUT procedures are variable and range from approximately 8 to 57% at 5 years of follow-up [5]. The problem may reflect persistent hypermobility or emergence of sphincter deficiency. This affects quality of life (QoL), ability to work and has substantial cost impact. Women with recurrent SUI commonly express desire to return to normal life, but they also wish to minimise the severity of surgery or complications. Up to 17% of women undergo a second operation for SUI within 10 years. The James Lind Alliance, a group of healthcare professionals and patients, identified this topic as a top 10 research priority in urinary incontinence [6].

This study ‘Proper Understanding of Recurrent Stress Urinary Incontinence Treatment in women’ (PURSUIT) is designed to help patients and doctors work out how to treat this common problem. It aims to establish whether surgical treatment is superior to endoscopic injections in terms of symptom severity at 1 year after randomisation in women with recurrent SUI. Study participants will be randomised between surgical and endoscopic interventions and the study is powered to ascertain clinically meaningful differences in symptom outcomes at 1 year after randomisation. The PURSUIT study addresses the research question “What is the best treatment for women with recurrent SUI after failed primary surgery?”.

Options to treat women with failed primary continence surgery used in the NHS include colposuspension, autologous fascial sling, AUS or endoscopic urethral bulking injections (bulking agents). MUT insertion is not currently available in the NHS.

The choice of surgical approach partly depends on the mechanism of the recurrent SUI, whether it is hypermobility or intrinsic sphincter deficiency. Colposuspension, autologous fascial sling or MUT are preferred by some surgeons for hypermobility, as these restore support for the urethra and bladder exit. Autologous fascial sling or AUS are believed to be more successful for women with recurrent SUI due to intrinsic sphincter deficiency, as they compress the urethra and thereby restore some resistance. For both mechanisms, endoscopy is a less invasive procedure than surgery. For SUI treatment with endoscopy, urethral bulking agents are injected into the urethra wall to reduce the size of the channel (also known as bladder neck injections). Examples of urethral bulking agents are Bulkamid®, Deflux® and Macroplastique®. There are a few substances marketed for urethral bulking, and a Cochrane review states that no clear-cut conclusions could be drawn from trials comparing alternative agents [7]. This review also suggested greater symptomatic improvement was observed with surgical treatments but set against likely higher risks.

### Objectives {7}

The primary objective of this study is to identify whether surgical treatment achieves a superior symptomatic outcome compared to endoscopic bulking injection(s) treatment at 1 year after randomisation in 250 women with recurrent SUI.

The secondary objectives of the study are to assess the longer-term (2 and 3 years after randomisation) clinical impact of the interventions on continence, the improvement of symptoms post-intervention, procedure/operative measures, sexual function, the safety of each intervention and the likelihood of re-treatment, the cost-effectiveness from both National Health Service (NHS) and societal perspectives at 1 year after randomisation and from a secondary care NHS perspective at 3 years after randomisation, women’s experiences of interventions and associated quality of life (QoL) and clinician’s views of interventions.

### Trial design {8}

**Fig. 1:**
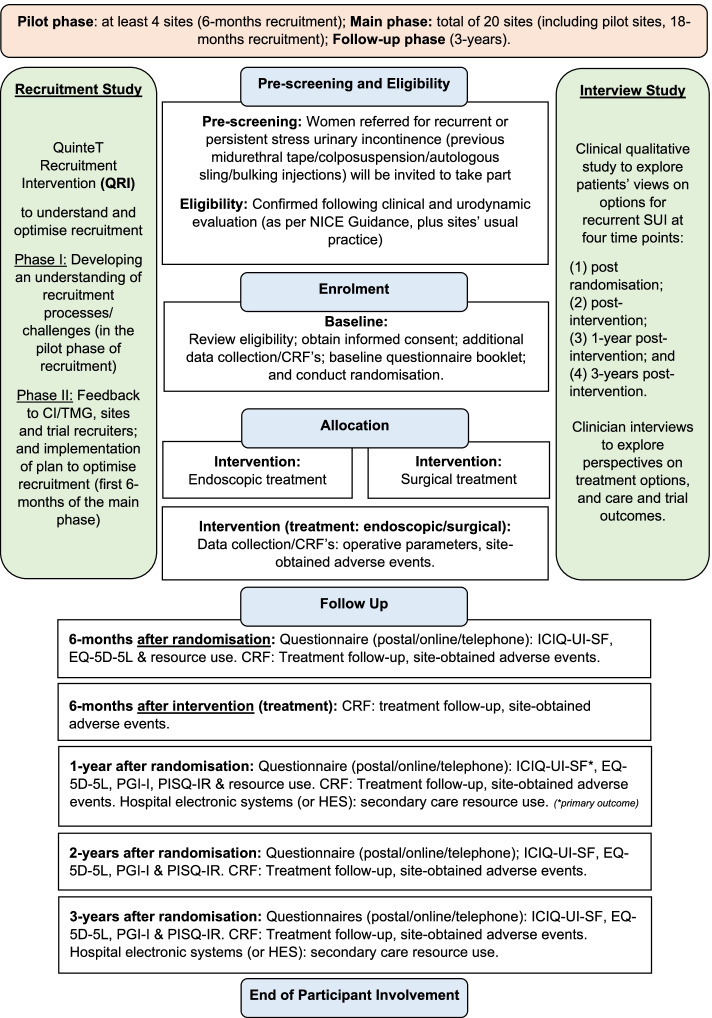
Trial flowchart

A two-arm randomised controlled trial in women with recurrent stress urinary incontinence comparing endoscopic intervention (urethral bulking injections) with a surgical intervention (current NHS options) (Fig. 1, trial flowchart). The patient allocation ratio is 1:1.

#### Internal pilot

Participants will be recruited over a 2-year period and followed up for 3 years. The recruitment period incorporates an internal 6-month pilot phase to test that our assumptions about recruitment and delivery of the interventions are achievable. At least four sites will recruit during the pilot phase. Recruitment projections have been developed allowing for the staggered opening of sites, start up and seasonal effects. Based on the projections, it is expected that 24 women will be recruited by the end of the pilot phase. The COVID-19 pandemic has resulted in considerable disruptions to the planned study timeline; therefore, timelines will be reviewed regularly by the Trial Management Group (TMG) and Trial Steering Committee (TSC) and discussed with the funder if adjustments are needed.

#### Nested studies

##### Recruitment Study—QuinteT Recruitment Intervention (QRI)

The PURSUIT trial will employ an integrated QuinteT Recruitment Intervention aimed at optimising recruitment and informed consent [8, 9]. Recruitment challenges may arise in relation to identifying potentially eligible women, differences in levels of equipoise amongst clinicians and women’s preferences for surgery or endoscopy. There may also be organisational challenges in relation to how the treatments are operationalised within the trial context and with the integration of the trial into existing clinical practice across sites. The QRI is aimed at identifying and addressing such recruitment difficulties promptly [8, 10, 11]. In PURSUIT, the QRI will be carried out intensively in the internal pilot phase with lessons learnt used to sustain recruitment during the transition to the main trial phase, similar to other surgical trials where this has been successfully implemented [12, 13].

The QRI uses novel qualitative and mixed-method approaches pioneered during the NIHR HTA-funded ProtecT (Prostate testing for cancer and Treatment) study, later refined and applied to several other Randomised Control Trials (RCTs), leading to insights about recruitment issues and the development of recruitment strategies [9, 14, 15]. The QRI will proceed in two iterative phases.

- Phase I: Understanding the recruitment process and how it operates at clinical sites. A multi-faceted approach will be used to investigate site-specific or wider recruitment obstacles.
- Phase II: Development and implementation of recruitment intervention strategies. The QRI team, with the CI and TMG, will formulate a ‘plan of action’ to improve recruitment and information provision, based on the findings from phase I.

##### Interview Study—qualitative research to understand women’s and clinician’s attitudes

Qualitative interviews will be conducted with study participants and clinicians involved in the trial to explore recurrent SUI generally, the acceptability and attitudes to the proposed treatments and to improve understanding of the shorter- and longer-term outcomes. To our knowledge, no studies to date have explored patient or clinician views on endoscopic/surgical treatment options for recurrent SUI.

Participant interviews will focus on attitudes to, and experiences of, endoscopic and surgical interventions. Interviews will be conducted at four time points (baseline, 6 months and 1 and 3 years post-randomisation) to capture views and experiences over time. Clinician interviews will explore views on the interventions along with facets of trial participation. Participants will be sampled purposively to ensure diverse participant characteristics are included. Data will be analysed using a thematic approach and findings will be used to interpret wider trial findings.

## Methods: participants, interventions and outcomes

### Study setting {9}

This trial will be delivered in a secondary care setting across (at least) 20 NHS urology and urogynaecology referral units in the United Kingdom (UK). Participating phase I and II sites will be listed on the PURSUIT study website [16].

### Eligibility criteria {10}

#### Subject population

Women with recurrent or persistent SUI.

#### Inclusion criteria

- Adult women (≥18 years) with bothersome SUI symptoms after primary SUI surgery (including bulking injections)
- Urodynamics to confirm recurrent or persistent SUI
- Patient willing to consider interventional therapy
- Patient willing to be randomised and willing to give consent

#### Exclusion criteria

- Predominant urgency incontinence
- Pelvic organ prolapse (POP) more than or equal to stage II
- Relevant neurological disease, disease, such as a stroke, multiple sclerosis, Parkinson’s disease, or spina bifida (diabetes mellitus is not an exclusion criterion unless it is causing diabetic neuropathy)
- Being treated for gynaecological or bladder cancer
- Unresolved mesh exposure from previous MUT
- Current pregnancy
- Urethral diverticulum
- Recent pelvic surgery (for example, POP repair, stress incontinence surgery and hysterectomy within the last 6 months)
- Participation in another study that might influence results or increase patient burden
- Unable to give informed consent/complete assessments
- Previous artificial urinary sphincter surgery

#### Site selection

Sites will be selected based on their referral populations and research capacity and capability.

#### Delivery of interventions

All professionals involved in delivery of the interventions will already be fully trained in the procedures, as these are specialist units recognised by subspecialist professional bodies (The British Society of Urogynaecology (BSUG), The British Association of Urological Surgeons (BAUS), Section of Female, Neurological and Urodynamic Urology). We will rely on quality-of-service delivery as scrutinised by the local continence multidisciplinary team (MDT, or equivalent) process.

### Who will take informed consent? {26a}

Due to the variation in patient pathways at each study site, recruitment arrangements will be individualised according to local practice.

#### Pre-screening and eligibility

Previous assessment results of women attending urology/urogynaecology clinics will be reviewed to determine eligibility. Patient notes and urodynamic unit clinical reports for women with previous midurethral tape/colposuspension/autologous sling/bulking injections referred for recurrent or persistent stress urinary incontinence will be assessed at sites by the clinical research team.

When a woman presents with symptoms suggestive of recurrent SUI, diagnostic testing to confirm urodynamic stress incontinence using standard approaches according to NICE will be considered [4, 17]. This testing will be done as part of their routine NHS clinical care. If a woman’s previous SUI surgery was midurethral tape, it will be considered whether it is possible that she might have a tape exposure (for example by physical examination, and consideration of whether cystoscopy is appropriate). If SUI is confirmed and there is no perceived risk of midurethral tape exposure being present, then the patient will be invited to participate.

Sites may also recognise other opportunities and methods for identifying potentially eligible women which should be utilised to minimise disruption to routine practice and involvement for the patient (e.g. during a routine clinical appointment/physiotherapy appointment/urodynamic assessment/multidisciplinary team (MDT) meetings).

Staff at all sites are asked to complete a trial-specific screening log for all potentially eligible women and provide confirmation of the patient’s outcome for the study; this will be one of three main outcomes: (1) patient confirmed as ineligible, (2) patient was eligible but declined to take part and (3) patient was eligible and consented to take part. Where possible, screening logs will include reason(s) for non-participation to ensure that participants are not approached more than once and to highlight patients who are willing to be contacted in the future if they were not able to participate when first approached (e.g. due to an acute intercurrent illness at that time). Sites will provide the central trial team (at the University of Bristol (UOB)) with a copy of their screening logs on a monthly basis, for monitoring purposes.

#### Invitation to participate

Potentially eligible women will be provided with the main study Participant Information Leaflet (PIL), and the Qualitative Studies PIL (covering the Recruitment Study and Interview Study), if appropriate. Site staff will follow up patients, either face-to-face or remotely, to discuss the study, to answer any questions they may have and to see if they would like to take part; this follow-up should be after at least 24 h and (ideally) within 8 weeks of the initial invitation. If the patient is eligible and would like to take part, written informed consent will be obtained. If the patient is identified during a clinical appointment, informed consent may be taken during that appointment (i.e. before the patient has had 24 h to consider the study), but site staff must follow up with the patient after 24 h has passed to ensure they are happy to continue *before* they are randomised.

Women who are eligible but decline to take part in the study may be asked to consent to being contacted for a qualitative research interview (Recruitment Study) to explore reasons for non-participation. Sites are expected to update patient medical notes indicating that the patient declined to take part in this study, providing study details (title), date, and any reason(s) if provided.

Staff (healthcare professionals, recruiting staff or TMG members) will be given the appropriate information leaflet inviting them to take part in either the Recruitment Study and/or the Interview Study.

#### Informed consent

##### Main study consent

Written informed consent will be obtained from all patients who are deemed eligible and agree to take part in the study. Consent may be obtained face-to-face (for example, during a clinical appointment or at a study-specific baseline visit), or remotely during a clinical, or study-specific, consultation which can be conducted via any method of contact employed/supported by the local NHS trust at the time. Consent for the study will be taken by a member of the site research team (a consultant, research nurse or trained and authorised delegate).

Written informed consent to take part may be obtained in the following ways:

- Written consent form—a study-approved paper (wet ink) consent form signed by the patient during a face-to-face consultation
- eConsent form—a study-approved (Health Regulatory Agency (HRA) and Medicines and Healthcare products Regulatory Agency (MHRA)-compliant) online eConsent form signed (electronically) by the patient during a remote or face-to-face consultation.
- Verbal consent form, followed by written consent form or eConsent form—a study-approved verbal consent form completed by the researcher during a telephone or video consultation with the patient.

##### Recruitment Study (QRI) consent

Written consent (paper or eConsent) for audio-recording/observing recruitment discussions will be obtained by research nurses (or other recruiting staff) from patients during their consultation when the PURSUIT trial is first discussed. If patients have not read the Qualitative Studies PIL in advance of their consultation, the recruiter will obtain verbal consent for the discussion to be audio-recorded, the PIL will be provided to patients at the end of the consultation and written consent will be obtained afterwards. If patients choose not to provide written consent, the recording made from their initial discussion will be deleted, and no further recordings will be made. Consent to be contacted about taking part in a research interview for the Recruitment Study is also included on the consent form.

Research nurses or the QuinteT researcher will obtain written consent from staff using a paper or online eConsent form. A one-off staff consent will cover all appointments, interviews and observations of meetings throughout the study.

##### Interview Study consent

Informed consent to be contacted about taking part in the Interview Study will be sought at the time of main study consent for patients, referring to interviews at a number of time points. After discussing concerns or questions, verbal informed consent will be requested and audio recorded at the point of interview. Participants will be informed that non-participation or withdrawal from the Interview Study will not affect their involvement in the main study. Verbal consent from clinicians will be requested and audio recorded.

### Additional consent provisions for collection and use of participant data and biological specimens {26b}

The approved PIL(s) and study consent form(s) include details about the collection and use of participant data. The consent form refers to the possibility of long-term follow-up and being contacted about other research if the participant is willing/invited.

(Consent for the collection and use of biological specimens is not applicable in this study as no biological specimens are collected).

## Interventions

### Explanation for the choice of comparators {6b}

Options to treat women with failed primary continence surgery are described below.

- *Autologous fascial sling*: a strip of the patient’s own tissue (fascia) is used to compress the urethra.
- *Colposuspension*: the anterior vaginal wall is repositioned to support the urethra.
- *Artificial urinary sphincter*: an implanted cuff is used to compress the urethra to keep the woman continent. The compression can be released by pressing on a component in the vaginal labium so the woman can pass urine when she wants to.
- *Endoscopic bulking injections*: a cystoscope is used to guide injection of bulking agents to the urethra, to enhance its ability to close effectively.

Early feedback from our Patient Advisory Group (PAG) and women affected by recurrent SUI was pivotal in establishing the design of the PURSUIT study. It was clear from discussions between patients and the clinical applicants that randomising women to specific surgical procedures was not an acceptable approach, an opinion echoed subsequently by the Research Ethics Committee (REC) during their review of the study. Each surgical operation has different implications and consequences and the impact on patients is highly individual. Furthermore, the surgery chosen should be appropriate for treating the underlying mechanism of the SUI identified in each individual woman (urethral hypermobility or intrinsic sphincter deficiency). It was concluded that endoscopic bulking injections could appropriately be compared against the other interventions (the surgical procedures).

Accordingly, the design of PURSUIT was determined following this important feedback. Women with confirmed recurrent SUI will be randomised to one of two treatment arms: endoscopic (bulking injections) or a surgical treatment.

### Intervention description {11a}

#### Assessment procedure

After women with a history of recurrent SUI have been identified, standard assessments as per NICE NG123 guidance [4] will be undertaken, including urodynamic testing, to confirm diagnosis. These assessments will also help to ascertain what type of surgery would be suitable for the patient, should she be randomised to the “surgical” treatment arm. Following these assessments, the patient is then randomised (allocated) to either the endoscopic (bulking injections) or the surgical treatment arm and will receive their intervention according to usual local practice.

#### Surgical treatment arm

The type of surgical intervention received by participants who are randomised to the surgical treatment arm will be decided following a detailed discussion between the patient and their surgeon (clinician), as per usual local practice. The PURSUIT study will not impose a specific surgical procedure upon patients. Surgery options available for recurrent SUI are autologous fascial sling, colposuspension and AUS. Currently, there are specific rules from the NHS which regulate the use of mesh in vaginal surgery, including midurethral tapes. The rules relevant at the time will be used for anyone wishing to consider this type of surgery.

#### Endoscopic (bulking injections) treatment arm

Endoscopic urethral bulking agents to reduce the size of the channel (also known as bladder neck injections) are injected into the urethra wall under direct vision, using a cystoscope. Examples of urethral bulking agents are Bulkamid®, Deflux® and Macroplastique®. The PURSUIT trial does not impose which urethral bulking agent(s) should be used; sites should use their usual urethral bulking agent(s). In the endoscopic arm, repeat injections are permitted.

### Criteria for discontinuing or modifying allocated interventions {11b}

Participants can choose to withdraw for any reason, at any time, during their involvement in the trial. Participants can withdraw from complying with the allocated trial treatment or providing data to the trial without affecting their usual care. In both cases, efforts will be made to report the reason for withdrawal as thoroughly as possible in a study-specific form. If a participant wishes to withdraw from receiving the allocated trial treatment, efforts will be made to continue to obtain follow-up data, with the permission of the patient or family as appropriate (including access to medical notes/databases). In the event the clinician feels it is unsafe for the participant to continue in the study, he/she can withdraw the participant from the study.

If a delay has occurred between confirmation of eligibility and the delivery of the allocated intervention, site staff will ensure eligibility is still valid before proceeding. If the participant is no longer eligible, for example due to a change in symptoms, they would be withdrawn from the study.

There should be no cross-over of patients from their randomised treatment allocation to the alternative treatment until after the primary outcome is recorded (1 year post randomisation). However, this is guidance only and cannot be imposed.

### Strategies to improve adherence to interventions {11c}

Study sites are asked to monitor and record all treatments that a participant receives; if cross-over of patients from their randomised treatment allocation to the alternative treatment arm does occur, then details, including reason(s) why, will be recorded in the study-specific case report forms.

### Relevant concomitant care permitted or prohibited during the trial {11d}

Participants will receive all standard care required during their participation in the trial, as per usual clinical practice.

### Provisions for post-trial care {30}

The interventions offered in this trial (both the operations in the surgery arm, and the bulking injections in the endoscopic arm) are part of standard NHS care. Accordingly, the patients will continue with the NHS standard care for their condition after the end of the research.

### Outcomes {12}

#### Primary outcome measure

The primary outcome is the patient-reported outcome measure (PROM) of continence using the International Consultation on Incontinence Questionnaire - Urinary Incontinence - Short Form (ICIQ-UI-SF) at 1 year after randomisation.

#### Secondary outcome measures

- Longer term clinical subjective measure of continence at 6 months and 2 and 3 years post randomisation (ICIQ-UI-SF)
- Improvement of symptoms post-intervention at 1, 2 and 3 years post randomisation (Patient Global Impression of Improvement (PGI-I) questionnaire)
- Procedure/operative assessment measures collected at the time of intervention and at 6 months post intervention: procedure/operation time, estimated blood loss, hospital stay, return to normal activity
- Assessment of sexual health at 1, 2 and 3 years post randomisation (Pelvic Organ Prolapse/Urinary Incontinence Sexual Questionnaire-IUGA Revised (PISQ-IR))
- The safety of each intervention (adverse events, AE) and the likelihood of re-treatment assessed at intervention, 6 months post intervention and 6 months and 1, 2 and 3 years post randomisation
- Cost-effectiveness from NHS and societal perspectives at 1 year post randomisation in terms of quality-adjusted life years (QALYs) and ICIQ-UI-SF at 1 year, and from a secondary care perspective in terms of QALYs at 3 years post randomisation. QALYs to be calculated from the EuroQol 5-Dimension 5-Level (EQ-5D-5L). Secondary care resource use to be abstracted from Trust electronic systems (or HES) at 1 and 3 years post randomisation, other resource use to be collected by questionnaires at 6 months and 1year post randomisation.
- Patient experiences of the intervention at 6 months and 1 year and 3 years post intervention (qualitative interviews with patients)
- Clinician views of the intervention at, or around, baseline (qualitative interviews with clinicians)

### Participant timeline {13}

The participant timeline is shown in Table 1.

**Table 1:** Participant timeline

	Pre-baseline	Baseline	Treatment	Post-treatment				Post-randomisation			
				3–6 months	6 months	1 year	3 years	6 months	1 year	2 years	3 years
**Pre-screening and eligibility**	X										
**Informed consent**		X									
**Randomisation**		X									
**Interventions**			X								
**Data collected by site staff**											
- Case report forms		X	X		X			X	X	X	X
- Adverse events			X		X			X	X	X	X
**Participant Questionnaires**											
- ICIQ-UI-SF		X						X	X	X	X
- PISQ-IR		X							X	X	X
- EQ-5D-5L		X						X	X	X	X
- PGI-I									X	X	X
- Non secondary care resource use								X	X		
**Secondary care resource use**									X		X
**Interview Study**											
- Qualitative interview (patients)		X		X		X	X				
- Qualitative interview (staff)		X									

### Sample size {14}

To inform our calculations we reviewed the literature using the ICIQ-UI-SF. A recent study of women with SUI suggested that the minimum clinically important difference was −5 when using anchor-based methods, and −2 with distribution-based methods [18]. We felt that the conservative estimate of −2 (equivalent to a difference of 0.5 standard deviation (SD)) was an important difference. To allow for the possibility that 5% of women randomised to surgery instead receive endoscopic treatment before 1 year, we reduced difference to detect to −1.9. Thus we estimate that we need to recruit 250 women (125 in each treatment arm) to detect a difference in mean ICIQ-UI-SF at 1 year of 1.9 (assuming common SD of 4.1; in line with the assumptions made in the study by Sirls et al. [18]) with 90% power and a significance level of 5%. This includes an inflation factor accounting for 20% loss to follow-up, as we are using a PROM as the primary outcome.

### Recruitment {15}

Participants will be recruited initially from 20 NHS urology and urogynaecology referral units in UK hospitals. If necessary, to reach the target sample size, the study will be opened at more referral units.

Assignment of interventions: allocation

### Sequence generation {16a}

The randomisation sequence will be generated by the BTC using their established (proven) online randomisation system or automated telephone system. Patients will be randomised on a 1:1 basis to the “endoscopic” or “surgical” intervention (treatment) arm. The individual randomisation will be stratified by site.

### Concealment mechanism {16b}

Site staff will only inform participants of their allocated intervention after the participant has completed and returned their baseline questionnaire to ensure responses are not biased by the patient’s knowledge of their allocation.

### Implementation {16c}

If study interventions are proceeding as usual (according to routine clinical pathways and timings), randomisation and data collection will be done when informed consent is obtained. If delivery of study interventions is paused, or there are considerable delays, due to the COVID-19 pandemic, patients may provide informed consent to take part, but randomisation and baseline data collection will not be undertaken until treatment interventions can proceed. As soon as treatment interventions can proceed, site staff will ensure eligibility and consent are still applicable and valid before conducting randomisation and collecting baseline data. Previous urodynamic results (used for initial confirmation of study eligibility) can be used providing that the doctor considers that they are still relevant, i.e. the tests were conducted since the woman’s last procedure and her symptoms have not changed since then.

#### Randomisation

A local research nurse or trained delegate (or member of the central trial team) will randomise patients using the online or telephone randomisation system. Randomisation will only be done after eligibility has been confirmed, written consent obtained and the patient has had at least 24 h to consider the study information and had any questions answered. Randomisation can be conducted during a face-to-face consultation (clinical appointment or study-specific visit) or be completed remotely. Site staff will inform participants of their treatment allocation during or after the consultation. Once a participant has been randomised, they are ‘enrolled’ in the study, they will be added to the appropriate procedure waiting list (as per usual local clinical practice) and treatment (intervention delivery) can proceed. Site staff will notify the participant’s General Practitioner (GP) that their patient has entered the trial.

### Assignment of interventions: blinding

#### Who will be blinded {17a}

Due to the nature of the interventions, participants and those administering the interventions will not be blinded to group allocation nor will the supporting clinical and site staff, to ensure relevant data collection. Two statisticians based at the UOB will support this trial. The senior statistician co-applicant will be blinded throughout the trial. The second trial statistician will perform all disaggregated analyses according to a pre-specified statistical analysis plan (SAP) and will attend closed Data Monitoring Committee (DMC) meetings as required. The health economist(s) will be blinded when cleaning data, but unblinded when conducting the analysis. Other members of the study team will remain blinded to aggregate data. The Study Manager and administrative staff will likely be unblinded to individual-level data to enable appropriate data collection.

#### Procedure for unblinding if needed {17b}

Not applicable. Only the senior statistician will be blinded throughout the trial.

### Data collection and management

#### Plans for assessment and collection of outcomes {18a}

Table 1 shows the participant timeline detailed data collected at each study time point. Baseline data will be collected after the patient has provided written informed consent, when their treatment intervention can proceed and as close to randomisation as possible. Wherever feasible, baseline data is collected by the research nurse, or other delegated site staff member during a face-to-face (or remote) consultation with the participant. Participant questionnaires at 6 months, 1, 2 and 3 years after randomisation will be sent by, and returned to, the central trial team. Participants can choose to complete questionnaires electronically or on paper. Site staff complete study-specific case report forms (CRFs) at baseline, the time of treatment, 6 months post-treatment and at 6 months and 1, 2 and 3 years after randomisation. Data are entered directly into paper questionnaires and CRFs and sent securely (by post or electronically via secure email transfer) to the central trial team for entry into the trial specific database.

### Validated participant questionnaires

The following validated questionnaires for patient-reported outcome measures (PROMs) will be used to minimise measurement bias. Table 1 shows the participant timeline details which PROMs are completed at each study time point.

- International Consultation on Incontinence Questionnaire-Urinary Incontinence Short Form (ICIQ-UI-SF) [19]. The questionnaire is used for evaluating the frequency, severity and impact on QoL of urinary incontinence in men and women across the world. It is also used to screen for incontinence, to obtain a brief yet comprehensive summary of the level, impact and perceived cause of symptoms of incontinence and to facilitate patient-clinician discussions.
- Pelvic Organ Prolapse (POP)/Urinary Incontinence Sexual Questionnaire, IUGA-Revised (PISQ-IR) [20]. This validated evaluation tool is used to assess sexual function in women with pelvic floor disorders.
- EuroQol Group’s 5-dimension health status questionnaire EQ-5D-5L [21]. A standardised instrument to measure generic health and the resulting profile is used to calculate QALYs.
- Patient Global Impression of Improvement (PGI-I) has been validated for use in females with urinary incontinence [22].

### Case report forms (CRFs)

A research nurse, or other delegated site staff member, will complete a study specific CRF at each appropriate study time point (see Table 1 participant timeline).

#### Baseline

The baseline CRF includes:

- Patient contact details, including email address
- NHS/CHI number
- Patient demographics, including date of birth and ethnicity
- GP contact details
- Charlson Comorbidity Index data
- Record of other diagnostic assessments (e.g. flow rate test/urodynamics/cystoscopy)
- Current medications (including whether on topical oestrogen therapy)
- Parity
- Previous pelvic surgery and dates
- Anticipated cause of SUI (hypermobility/intrinsic sphincter deficiency/both/not diagnosed)

#### Time of treatment

Research staff at each site will record procedure/operative data in the study “peri-operative CRF”; this refers to the period from when the participant is admitted to hospital to undergo treatment (intervention) for their SUI (surgery or endoscopic urethral bulking injection), through to when they are discharged for their primary treatment (intervention). The peri-operative CRF includes:

- Date of admission
- Height, weight, and body mass index (BMI)
- American Society of Anaesthesiologists (ASA) physical status classification
- Date of procedure (if different from admission)
- Hospital where procedure took place
- Name of surgeon
- Treatment/Operative procedures
  - ◦ Endoscopic arm: type of urethral bulking agent(s) used
- Complications
- Details of catheterisation
- Transfusion
- Post void residual volume (PVR)
- Adverse events
- Date of discharge

#### Six months after treatment

- Adverse events
- Endoscopic arm only: re-intervention (i.e. was a repeat injection needed?)

#### Six months and 1 year, 2 years and 3 years after randomisation

- Complications of treatment (if applicable)
- Details of catheterisation (status/duration/other details)
- Have any other treatment procedures (interventions) taken place since initial treatment? If so, relevant (what/when) details of what and when.
- Adverse events
- Endoscopic arm only: re-intervention (i.e. was a repeat injection needed?)

### Health economic data collection

Participant questionnaires at 6 months and 1 year after randomisation will also include questions relating to community-based NHS resource use, patient costs (e.g. incontinence pads), time off work and return to normal activities.

Secondary care resource use data will also be collected from each study site at 1 and 3 years after randomisation, via the hospital electronic systems (medical record abstraction), and will include inpatient and day case admissions, outpatient visits and procedures and accident and emergency attendances. Information from the hospital systems will be requested in the form of Healthcare Resource Group (HRG) codes for inpatient stays, day cases and outpatient procedures. For outpatient visits, currency codes will be requested to designate the type of outpatient appointment (e.g. consultant face-to-face) and a service code to identify the clinical specialty. For accident and emergency visits, a currency code will be requested to indicate the intensity of treatment and a service code to indicate whether the patient was subsequently admitted to hospital. If it is not possible to obtain this information from the hospital trusts, an application to NHS digital to obtain Hospital Episode Statistics (HES) database will be made.

### Qualitative data collection

#### Recruitment Study phase I—understanding recruitment

Data for phase I of the Recruitment Study will be collected as below.

- *Mapping of eligibility and recruitment pathways*

Detailed eligibility and recruitment pathways will be compiled for clinical sites, noting the point at which women receive information about the trial, which members of the clinical team they talk to and the timing and frequency of appointments. These will be compared with details specified in the trial protocol and pathways from other sites to identify those that are potentially more/less efficient. Screening log data will be collected at sites detailing the numbers of screened, eligible, approached and randomised patients (SEAR approach) [23]. Adherence to treatment allocation amongst those randomised and reasons for non-participation amongst decliners will also be noted. These will help identify points at which women do not continue with recruitment and be considered in relation to estimates specified in the grant application/study protocol.

(b) *Audio-recording and observations of recruitment discussions (at four pilot sites only)*

Scheduled face-to-face appointments or remote consultations (e.g. telephone or video-call) during which the trial is discussed with the patient will be routinely audio-recorded (and if necessary, also observed) with written consent. Audio-recordings will be made using an encrypted device. The audio-recordings will be used to explore information provision in relation to key study concepts and treatment options, recruitment techniques, management of patient treatment preferences, and randomisation decisions to identify recruitment difficulties and improve information provision. Audio-recordings will be collected by trial staff across sites and transferred to/from the UOB through UOB-approved secure data transfer facilities or encrypted flash drives/memory cards that adhere to NHS Trust policies.

(c) *In-depth interviews*

Semi-structured interviews will be undertaken with three groups:

- Members of the TMG, including the Chief Investigator (CI) and those involved in the design, management and leadership of the trial.
- Clinicians or researchers who are involved in the patient pathway and trial recruitment (at four pilot sites only).
- If necessary, eligible women who have been approached to take part in the trial (at four pilot sites only).

Interviews with members of the TMG and clinicians or researchers (recruiters) will explore their perspectives on the RCT, and where relevant, their experiences of recruitment. Interviews with eligible women will explore views on the presentation of study information, understanding of trial processes (e.g. randomisation) and reasons underlying decisions to accept or decline the trial. Professionals as well as women will be purposefully sampled, to build a sample of variation on the basis of characteristics such as professional expertise, trial recruitment experience and study site or age and the final decision about trial participation (i.e. accept or decline), respectively. The number of interviews will be guided by data saturation (when no new information is forthcoming) and other considerations (e.g. timing of interviews).

Interviews will take place at a mutually convenient location, in a suitably private and quiet setting or participants will be offered the option to be interviewed over the telephone or via a video-call. Interviews will be audio-recorded using an encrypted device (as described above). UOB ‘lone researcher’ safety policies will be adhered to for any interviews taking place in non-public settings (e.g. participants’ homes).

(d) *Observation of TMG and investigator meetings*

The QRI researcher may observe and make detailed notes of study meetings to gain an overview of trial conduct and overarching challenges (logistical issues, etc.). These meetings may be audio-recorded with informed consent.

(e) *Study documentation*

The PIL and consent form will be contrasted with the interviews and recorded appointments, to identify any disparities or improvements that could be made.

#### Recruitment Study phase II—development and implementation of recruitment intervention strategies

The QRI team, with the CI and TMG, will formulate a ‘plan of action’ to improve recruitment and information provision, based on the findings from phase I. Generic forms of intervention may include ‘tips’ documents that provide suggestions on how to explain the trial design and processes. Supportive and responsive feedback will be a core component of the plan of action, with the exact nature and timing of feedback dependent on the issues that arise. Site-specific feedback may cover institutional barriers, while multi-site group feedback sessions may address widespread challenges that would benefit from discussion. All group feedback sessions will be aided by displaying anonymised data extracts from interviews and audio-recorded consultations. Individual confidential feedback will also be offered, particularly where recruiters experience specific difficulties, or where there is a need to discuss potentially sensitive issues. Investigator meetings/teleconferences and site visits from the CI/TMG members may also be employed to discuss technical or clinical challenges (e.g. discomfort surrounding eligibility criteria).

QRI phases I and II are likely to overlap. New avenues of enquiry will emerge throughout the conduct of the QRI (e.g. in feedback meetings), and rigorous monitoring of screening logs before and after interventions may indicate a need for further investigations (phase I) or intervention (phase II).

Recruitment figures (numbers of screened, eligible, approached and randomised women) will be regularly monitored. Continued targeted investigation of recruitment issues and delivery of feedback/training will be undertaken as necessary, with particular focus on changes in recruitment practice before and after the intervention.

#### Interview Study

A standardised approach will be employed to explore the areas described below in accordance with published qualitative research methods. Interviews will be carried out by an experienced qualitative researcher and will be conducted either face-to-face or over the telephone. Interviews will be semi-structured and follow a topic guide informed by a literature review and discussion between study researchers and encourage participants to discuss their perspectives. Approximately 40 participants (20 in each intervention arm) will be interviewed at baseline and again at follow-up time points during the main trial to explore the intervention trajectory. Approximately 10 clinicians will be interviewed in order to capture sufficient viewpoints to evaluate the clinical perspective on both intervention arms involved.

Participant interviews:

i. At baseline (following randomisation): Health-seeking drivers, previous treatment experience and perceptions of effectiveness, product usage, perspectives on both endoscopic and surgical treatment options—what would women like/expect to be offered, expectations regarding outcomes and determinants of satisfaction.
ii. At follow-up (3 to 6 months following delivery of the treatment): Perspective on treatment received; positive and negative aspects of the treatment, including pain, post-procedure recovery, associated symptoms, symptom improvement or deterioration, new onset symptoms; return to activities and daily life impact, product usage.
iii. At long term follow-up (12 and 36 months following delivery of the treatment): Long-term perspective on treatment received; symptom status; comparison with expectations, positive and negative aspects of the treatment; product usage; desire for further treatment; requirement for coping strategies; would the participant advocate the procedure; satisfaction with symptom status.

Clinician (Urologist/Urogynaecologist) interviews:

i. Perspectives on the different methods of treatment and available options within those groups; treatment preferences; reasons for endoscopic versus surgical treatment decisions; technical aspects of the procedures.
ii. Perspectives on care outcomes—symptom status, length of recovery, long-term results, complication rates.
iii. Perspectives on trial outcomes—women may receive different treatments than the clinician would usually advocate.

Theoretical purposive (non-probability) sampling will be used to ensure the diverse characteristics of the population are sampled (e.g. participants varying in age, clinical history, intervention arm, duration of symptoms). Geographical distribution will also be factored to ensure representation of varied populations. Sampling and analyses will continue in iterative cycles until no new themes are emerging and established themes cease evolving (data saturation).

### Plans to promote participant retention and complete follow-up {18b}

We will take active measures to minimise loss of women from the trial. Questionnaire completion will be followed up and managed by the central trial team using a central administrative database to notify the team when questionnaires are due. If a participant fails to return a questionnaire, a total of up to four reminders per time point (either telephone, email or postal) can be actioned by the site team or central trial team. In the case of a missing questionnaire, the response to the questionnaire can be collected from the participant over the telephone to reduce loss to follow-up. Multiple options are available to participants for them to complete their questionnaires, this ensures that the questionnaires are easily accessible to everyone (post/online/telephone). Other methods to promote participant retention will include obtaining back-up ‘best contact’ addresses, contacting the participant’s GP practice to check their contact details on record are still valid [24] and using ‘thank you’ vouchers as retention incentives [25]. In addition, we may access centrally held NHS data, for example via the NHS Strategic Tracing Service in England and Wales, to find new addresses.

If a participant withdraws/is withdrawn from the study, the study team would retain, confidentially, any data collected up to the point of withdrawal for analysis. As advised in the PIL, we will continue to collect data from a participant’s electronic records unless they request otherwise.

### Data management {19}

#### Source data and documentation

Source data will consist of paper or electronic copies of the consent form, participant completed questionnaires, paper CRFs designed specifically for the study and audio-recordings of consultations and interviews. Where data is recorded first in the patient’s medical records that is, and will remain, the primary source data and specifically designed CRFs would be considered supplementary source data. When a participant consents to enter the trial, they will have a unique participant identification (ID) number allocated to them. PIs (or delegated member of staff) must keep records of all participating patients (sufficient to link records e.g. CRFs and hospital records), all original signed informed consent forms and copies of the CRFs.

#### Database platforms

Data, including participant’s personal data, will be entered directly into a password protected REDCap database by trained members of staff within the central trial team. REDCap is a secure, web-based electronic data capture (EDC) system designed for the collection of research data [26]. The system has been developed and supported by Vanderbilt University. BTC has set up its own infrastructure so that all systems are hosted at, and supported by, UOB. The database will be maintained on a SQL server database system within the UOB and will only be accessible to members of the research team. Any data stored on laptops will be encrypted. Any information that is analysed or transferred outside the UK will be anonymised. Participants will be informed via the PIL that personal information such as their name, email address and phone number will be stored on the secure database with the central trial team.

Administrative and clinical study data will be stored in separate REDCap instances. A Relational Database Management System will be used to provide integration services between administrative and clinical databases. The administrative data will be kept in a secure database that is only accessible from within the UOB firewall. All users will require (at least honorary) contracts with UOB to access it. Anonymised clinical data is linked by the study participant ID to the administrative data. Email addresses are collected as they are essential for providing participant questionnaires electronically. The ‘Email Address’ field is flagged as an identifier and not included in the export for the statistician, so the data set can be considered pseudonymised at export and doesn't need further processing.

#### Data storage and handling

North Bristol NHS Trust and the Bristol Trials Centre (University of Bristol) are joint data controllers for the PURSUIT Trial. Data will be held at the UOB and will conform to the UOB Data Security Policy. Data will be collected and retained in accordance with the Caldicott Principles, UK Data Protection Act (DPA) 2018 and General Data Protection Regulation (GDPR). Personal data (e.g. name and address, or any data from which a participant might be identified) will not be kept for longer than is required for the purpose for which it has been acquired. All electronic data files will be saved in a secured computer and to a password protected UOB network space, in accordance with the UOB’s data security policies.

#### Access to data

For monitoring purposes, the CI will allow monitors from the sponsor (or delegate), persons responsible for the audit, representatives of the REC and other Regulatory Authorities to have direct access to source data/documents. The Data Manager (in collaboration with the CI) will manage access rights to the data set. Prospective new users must demonstrate compliance with legal, data protection and ethical guidelines before any data are released.

#### QRI and Interview Study data

Where applicable, site staff will be asked to set up an audio-recorder during recruitment discussions with potential participants. Audio-recorders will be stored securely at sites in a locked drawer/cabinet when not in use and returned to the central trial team securely at the end of the study. Audio-recordings of appointments in which the trial is discussed will be held on the encrypted digital audio-recorder and regularly transferred to the UOB through approved secure data transfer facilities and/or encrypted flash drives/memory cards that adhere to NHS Trust policies. Interview data captured on an audio-recorder will be uploaded to a secure, password-protected UOB server as soon as possible after each interview.

All audio-recorded data will be stored on a password-protected computer maintained by the UOB. Audio recordings of interviews and appointments will be transcribed verbatim in full or in parts (targeted) by a UOB employee or UOB-approved transcription service, with required confidentiality agreements in place. Audio-recordings and transcripts will be labelled with a unique identification number, edited to ensure anonymity of respondents and stored securely adhering to the University’s data storage policies. Interview data will be managed using NVivo software (QSR International) [27].

Anonymised quotations and parts of voice-modified recordings may be used for training, teaching, research and publication purposes for this and future studies. Anonymised transcripts may be made available to other researchers who secure the necessary approvals for purposes not related to this study, subject to individual written informed consent from participants. At the end of the study, anonymised data (including transcripts of audio-recordings) will be stored in a secure research data storage facility, alongside the other study data.

#### Archiving and destruction of trial materials

An archiving plan will be developed for all trial materials. Data will be held in compliance with the sponsor’s standard procedures. All research data will be retained in a secure location during the conduct of the trial and for at least 5 years after the end of the trial. Data will be kept at the UOB for this time and, at the end of the archiving period, will be destroyed by confidential means with the exception of a final trial dataset which will be made available for data-sharing purposes. The approval of NBT as owner of data and Study Sponsor, as well as the CI, will be sought prior to destruction of the data. Participating sites will be responsible for ensuring that all study records held at site are archived appropriately when notified by the Sponsor or central trial team.

### Confidentiality {27}

When a participant consents to enter the trial, they will have a unique participant ID number allocated to them. Information capable of identifying individuals and the nature of treatment received will be held in the database with passwords restricted to trial staff. Information capable of identifying participants will not be removed from clinical sites apart from when sending data to the central trial team at the UOB by secure email transfer. Information capable of identifying individuals will not be made available in any form to those outside the trial, with the exception of NHS digital for linkage, or for inspection purposes by the sponsor or other regulatory authorities. Consent forms and clinical letters with personal identifiable data will be stored in a locked filing cabinet. Participant details will be anonymised in any publications that result from the trial.

### Plans for collection, laboratory evaluation and storage of biological specimens for genetic or molecular analysis in this trial/future use {33}

Not applicable, no biological specimens are collected for this trial.

## Statistical methods

### Statistical methods for primary and secondary outcomes {20a}

All analyses and reporting will be in line with CONSORT guidelines. Primary analyses will be performed on the intention-to-treat (ITT) basis, analysing women in the groups to which they were randomised. A full SAP will be developed and agreed by the TSC and DMC prior to undertaking analyses for the main trial and will be submitted as an update.

#### Summary of baseline data and flow of participants

Descriptive statistics will be used to summarise characteristics of patients and compare baseline characteristics between groups. Means and SDs will be used for continuous outcomes or medians and interquartile ranges if required for skewed data. Categorical variables will be summarised using frequencies and proportions. Baseline variables to be explored include those described in the ‘Plans for assessment and collection of outcomes {18a}’ section. Patient-reported outcome scores based on standardised questionnaires will be calculated based on the developers’ scoring manuals and missing erroneous items will be handled according to these manuals.

Secondary analyses will adjust for any prognostic variables showing a marked imbalance at baseline (ascertained using descriptive statistics).

#### Primary outcome analysis

The PROM, ICIQ-UI-SF at 1 year post-randomisation is the primary outcome. Comparisons between treatment arms will be made using a multivariable linear model with random effect for site to account for within-site correlation. The model will adjust for baseline ICIQ-UI-SF scores. The underlying assumptions of the model will be checked, and analyses adjusted accordingly.

#### Secondary outcome analysis

The secondary outcomes in this study explore the longer-term impacts of the intervention on self-reported and objective improvements in continence and sexual function. Continuous measures will be studied in the same manner as the primary outcome and ordered categorical variables will be studied using ordinal logistic regression. Where outcomes are measured at multiple time points post-randomisation repeated measures analyses will be used to examine whether treatment effects are sustained, diminished or emerged later. These will be investigated formally by introducing an interaction term between treatment arm and time. All models will adjust for the outcome at baseline.

Surgical outcomes will be described using descriptive statistics for those women allocated to the surgical arm and no formal comparisons will be made between surgeries.

### Interim analyses {21b}

Women will complete outcome measures at 6 months and 1, 2 and 3 years after randomisation. Analyses of the 6-month to 3-year follow-up data will be completed at the same time, as the 2- and 3-year follow-up data provides context for the primary outcome data at 1 year. An independent DMC will review confidential accumulating data at its discretion, but at least annually. No interim statistical analyses by study arm are planned.

### Methods for additional analyses (e.g. subgroup analyses) {20b}

#### Subgroup analyses

We will conduct a small number of pre-defined subgroup analyses to assess whether the difference in ICIQ-UI-SF at 1 year between the two treatment arms differed according to baseline characteristics including age. Effect modification will be assessed by including an interaction term in the regression model and formal tests of interaction will be performed. These analyses will be outlined in detail in the SAP which will be agreed in advance by the TSC and DMC.

#### Adjusted analysis

All primary analyses will adjust for the outcome as measured at baseline. Secondary analyses will adjust for any prognostic variables demonstrating marked imbalance at baseline as determined using descriptive statistics.

#### Health economic evaluation

The base case cost-effectiveness analyses will be from an NHS perspective, comparing costs in relation to QALYs at 1-year follow-up. A societal perspective analysis at 1 year and a further NHS secondary care perspective analysis at 3 years, comparing costs in relation to QALYs, will also be conducted. Discounting for the 3-year analysis will be based on NICE recommended rates at the time, currently 3.5% for both costs and benefits. The relevant most up-to-date NHS reference costs will be used to value the information obtained from either the Hospitals’ costing systems or HES. Community-based NHS resource use, time off work and normal activities will be valued using routine data, e.g. Unit Costs of Health and Social Care; ONS Annual Survey of Hours and Earnings. Participant car travel will be valued using HMRC advisory fuel rates. All other travel costs and out of pocket expenditure will be valued as reported by the participants.

The EQ-5D-5L will be administered at baseline, 6 months and 1, 2 and 3 years after randomisation. These values will be transformed into utility scores using the recommended value set at the time of analysis, and individual QALYs will be calculated using the area under the curve approach.

At both 1-year and 3-year time points and for all perspectives, differences in mean costs and QALYs between the trial arms will be evaluated using appropriate regression techniques, adjusting for site and in the case of QALYs, baseline utility.

Incremental cost-effectiveness ratios will be calculated if no arm is dominant, i.e. more effective and less costly than the other arm. Incremental net monetary benefit statistics will also be produced over a range of willingness to pay thresholds for a QALY.

Additionally, at 1 year, a cost consequence analysis from an NHS perspective will be used to compare the differences in costs and the differences in ICIQ-UI-SF.

Uncertainty for all analyses will be addressed using cost-effectiveness acceptability curves and sensitivity analyses. A health economics analysis plan (HEAP) will be produced prior to analysis, in which sensitivity analyses will be outlined. These are likely to include different approaches to dealing with missing data, based on reasons why the data might be missing.

#### QRI data analysis

Analysis of QRI data will be led by the qualitative research associate with the guidance of the QRI lead researcher, with a sample of transcripts independently coded by both researchers. QRI transcripts will be analysed thematically using constant comparative approaches derived from Grounded Theory methodology [28]. Audio-recorded recruitment appointments and follow-up discussions will be subjected to content, thematic and novel analytical approaches, including aspects of targeted conversation analysis and appointment timing (the ‘Q-QAT method’) [29, 30]. There will be a focus on aspects of information provision that are unclear, disrupted, or potentially detrimental to recruitment and/or adherence. Key issues identified from the observation notes of appointments and TMG/investigator meetings will be considered alongside other qualitative findings. Findings from all sources will be drawn together in a descriptive account that will be presented to the CI/TMG and will guide development of the recruitment intervention plan (QRI Phase II).

#### Interview Study analysis

Interview transcripts will be analysed by the qualitative researcher on an ongoing basis in an iterative manner, according to principles of thematic content analysis. Recordings will be listened to, and transcripts read and re-read for familiarisation. Segments of text will be ‘coded’ by assigning descriptive labels. Codes will be grouped on the basis of shared properties to create themes and coded transcripts will then be examined and compared to inductively refine and delineate themes (constant comparison). A subset of interviews will be independently analysed by a second study researcher and coding discrepancies discussed to maximise rigour and reliability. Plausibility of data interpretation will be further discussed between the study team throughout the analyses. Descriptive accounts of the audio-recordings and interviews will be prepared.

### Methods in analysis to handle protocol non-adherence and any statistical methods to handle missing data {20c}

The primary analyses will be based on the observed data and a sensitivity analysis will be conducted where missing data are imputed using appropriate methods based on patterns of missingness. Data will be entered promptly, and data validation and cleaning will be carried out throughout the trial. Where spurious data are observed, values will be checked against available records.

### Plans to give access to the full protocol, participant-level data and statistical code {31c}

The full study protocol is available online via the NIHR funding award records [31] and via a link in the study International Standard Randomised Controlled Trials Number (ISRCTN) registry [32], both of which are publicly accessible. The datasets analysed during the current study will be available to other researchers through a data sharing agreement (DSA) and the statistical code will be available from the corresponding author on reasonable request.

## Oversight and monitoring

### Composition of the coordinating centre and trial steering committee {5d}

#### Coordinating centre

The central trial team will be based within the BTC, a United Kingdom Clinical Research Collaboration (UKCRC)-registered clinical trials unit and will support the delivery and conduct of the trial. The CI will take overall responsibility for managing the various components of the trial and will meet at least monthly with the leads for each component. The CI will be supported by the Trial Manager who will take responsibility for the day-to-day management of the trial with the Trial Administrator and the lead Research Nurse. The data management team will oversee all IT aspects of the study.

#### Trial Management Group (TMG)

A TMG will meet at least once each quarter in the first 2 years, then 6 monthly to review progress, with ad hoc meetings, as required. The TMG will have responsibility for the day-to-day management of the trial and will report to the TSC. It will be chaired by the CI and will consist of relevant co-applicants, including a Patient and Public Involvement (PPI) co-applicant and representatives from the Sponsor and BTC.

#### Trial Steering Committee (TSC)

Membership, responsibilities and reporting mechanisms of the TSC are detailed in the TSC charter. The TSC will make key decisions and recommendations during the trial to the TMG and notified to the funder.

The TSC will comprise of an independent chair plus three additional independent members (a clinician, a statistician and an independently nominated PPI representative). The independent members will cover expertise in statistics, trials, urology and urogynecology. The CI will also be a formal (non-voting) member of the TSC. Observers may also attend (including other members of the TMG or members of other professional bodies) at the invitation of the Chair. The TSC will meet for the first time by month 6 of the trial and then 6 monthly thereafter.

### Composition of the data monitoring committee, its role and reporting structure {21a}

The DMC will monitor accumulating trial data for quality, completeness and patient safety and will make recommendations to the TSC regarding ethical or safety issues which may require a protocol amendment or closure of the trial. The DMC will comprise an independent chair, two other independent members with expertise in trials and statistics, and gynaecology and urology and the CI. Responsibilities and reporting mechanisms of the DMC are detailed in the DMC charter which has been developed using the DAMOCLES charter for independent DMCs [33]. The DMC will meet once prior to recruitment of the first participant and again during years 2, 3, 4 and 5 prior to the TSC meeting, to review the AE data and any other ethical aspects that arise and will report to the TSC.

### Adverse event reporting and harms {22}

AE data are collected and assessed throughout an individual’s participation in the study as secondary outcomes. The PI at each participating site (or appropriate delegate) is responsible for categorising whether AEs are serious, expected and related. Serious and non-serious adverse events (S/AEs), reported by the participant or research teams, will be recorded, and reported in accordance with the Good Clinical Practice (GCP) guidelines and the Sponsor’s Research Related Adverse Event Reporting Policy.

#### Adverse event (AE)

An AE includes any untoward medical occurrence in a study participant administered an intervention which does not necessarily have a causal relationship with this treatment. In all instances, it will be up to the Principal Investigator (PI) of each participating site (or appropriate delegate) to determine whether the person’s change in health is related to the trial. AEs are not continuous and persistent disease or symptoms, present before the trial, which fail to progress, signs or symptoms of recurrent SUI or treatment failure.

#### Serious adverse event (SAE)

A SAE is any untoward medical occurrence that:

- Results in death
- Is life-threatening
- Requires inpatient hospitalisation or prolongation of existing hospitalisation
- Results in persistent or significant disability or incapacity
- Consists of a congenital anomaly or birth defect.

Important AEs that are not immediately life-threatening or do not result in death or hospitalisation but may jeopardise the participant or may require intervention to prevent one of the other outcomes listed in the definitions above, will also be considered serious. Medical judgement will be exercised in deciding whether an AE is serious in other situations.

#### Expected events

Due to the nature of the treatments for recurrent SUI, AEs are expected to occur throughout the course of the trial. Events that can be expected during/after any surgery, or within this patient population are listed below.

- Anaesthetic complications, e.g. stroke or cardiac events such as myocardial infarction
- Operative injury to adjacent structure
- Fistula
- Return to theatre
- ITU admission
- New urinary tract symptoms
- Urinary tract infection
- Wound infection
- Pelvic organ prolapse (POP)
- Urinary retention/catheterisation (intermittent self-catherisation (ISC) and indwelling)
- Pain
- Implant exposure (tape, AUS)
- Incisional hernia
- Deep vein thrombosis (DVT)/Pulmonary embolism (PE)
- Bleeding/haematoma/blood transfusion
- Chest infection
- New sexual problems e.g. dyspareunia
- Other infections (sepsis, septicaemia, abscess, respiratory)
- Inflammation, e.g. osteitis pubis
- Death

#### Reporting procedures

All adverse events (serious and non-serious) will be recorded in the participant’s medical notes and appropriate study CRF. SAEs will also be recorded in a study-specific SAE log which will be regularly reviewed by the DMC and Sponsor.

- Non-fatal expected SAEs and unexpected SAEs which are not causally related to the research procedures will not be reported to the Sponsor.
- Fatal expected SAEs and unexpected SAEs which are causally related to the research procedures (serious adverse reactions, SARs) will be reported to the study Sponsor within 24 h of staff becoming aware of the event. SARs will also be reported to the REC immediately.

The PI (or trained delegate) at each study site is responsible for identifying and categorising AEs, ensuring all SAEs are documented, reported and followed up appropriately. The CI is responsible for clinical oversight of the safety of patients participating in the trial including an ongoing review of the risk/benefit, categorising AEs where it has not been possible to obtain local medical assessment, reviewing all reportable SAEs and ensuring appropriate reporting of SAEs.

### Frequency and plans for auditing trial conduct {23}

The study will be monitored in accordance with the sponsor’s (North Bristol NHS Trust) Monitoring Standard Operating Procedure (SOP), which is consistent with the UK Policy Framework for Health and Social Care Research. All trial-related documents will be made available on request for monitoring and audit by North Bristol NHS Trust, the REC and available for inspection by other licensed bodies.

### Plans for communicating important protocol amendments to relevant parties (e.g. trial participants, ethical committees) {25}

The TSC and Sponsor will be required to approve any important amendments to the study protocol, prior to submitting to the REC/HRA for approval. Following HRA/REC approval, amendments will be shared with the research and development offices, PI’s and research teams at each study site in accordance with standard HRA guidance. Training support in any new processes will be provided to all site teams, where necessary. If an amendment affects trial participants in any way, they will be informed about the changes.

### Dissemination plans {31a}

The results of the study will be published in the academic press and on the Sponsor and UOB websites [16, 34]. All participants will be offered a lay summary of the main findings of the study. The trial results will also be presented at national and international conferences and disseminated to the national specialist bodies with responsibility for guiding clinical practice, policy matters, research priorities, governance and training in matters related to incontinence (BAUS and BSUG).

## Discussion

This article outlines a definitive two-arm multicentre RCT to compare endoscopic intervention (urethral bulking injections) with surgical interventions (colposuspension, autologous fascial sling, MUT (if allowed) or AUS) in adult women with recurrent SUI. The aim is to determine whether surgical treatment is superior to endoscopic bulking injections in terms of symptom severity 1 year after randomisation.

Here we discuss the key challenges encountered during the first 2 years of the trial and adaptations made to support study delivery.

### The impact of the COVID-19 pandemic

#### Site capacity

The study was open for just 10 weeks before recruitment was halted for 6 months due to the emergence of the COVID-19 pandemic in March 2020. Despite approval to re-start the trial in September 2020, the immense impact of the pandemic on the NHS is ongoing and will be long-lasting. The capacity of research and clinical teams to open the study and conduct trial activity over the past 2 years has been substantially reduced and some sites have had to withdraw from participation.

#### Low surgical prioritisation of the patient group

During the pandemic, the Royal College of Surgeons (RCS) implemented a clinical guide to surgical prioritisation, classifying all procedures from P1 (priority 1—immediate treatment) to P4 (priority 4—procedures to be performed in >3 months). All incontinence procedures, including those for recurrent SUI, are classified as the lowest priority (P4) as the condition is not life-threatening, but with the substantial backlog of operations, P4 currently means “delayed indefinitely”. Waiting lists for these treatments are now, at many hospitals, greater than 1 year, with some approaching 2 years. Many women with recurrent SUI have lived with symptoms, which severely affect their quality of life, for years and sometimes even decades. They have experienced an unsuccessful treatment, which can cause considerable distress and their ongoing symptoms affect their self-esteem, relationships, and their occupations. Some of these women have also undergone removal of vaginal mesh which may have caused their symptoms to deteriorate to a level worse than when they first sought treatment. Their symptoms are marginalised by this low prioritisation, which is a backward step, considering that it took years for incontinence to receive due recognition for its true impact on women. The issue is being escalated to the relevant bodies, with the aim of re-assessing and re-classifying prioritisation for this patient group.

#### Patient reluctance to seek further treatment

Women with recurrent SUI are often reticent to seek further treatment because of their experience of a previously failed procedure and some women have understandably lost trust in the medical community due to the widely reported potential complications of vaginal mesh surgery. During the pandemic, PURSUIT site teams have reported that fewer women than usual are being referred to secondary care for recurrent SUI treatment. This reduction may be indicative of the impact of the pandemic on primary care capacity but could also signify an additional reluctance of women to seek help from an already over-burdened and struggling health service. Furthermore, our patient and public involvement contributors have voiced their concerns about visiting a clinical setting during the pandemic, where there could be an increased risk of COVID transmission; thus, it is likely this may also be an influencing factor.

### Adaptations to study design and conduct

The timelines for study delivery have been significantly disrupted; thus, the pilot phase and main recruitment phases are now being conducted concurrently. Findings from the QRI sub-study have been used to develop and roll-out a comprehensive training package for recruiting sites. The training has provided further scope to identify recruitment barriers, while supporting and optimising recruitment where possible. Every stage of study delivery has been affected by the challenges described above, and where possible, resolutions have been implemented to address these.

#### Identification of sites

A number of pre-identified sites had reduced or no capacity to proceed with study set up during the pandemic. We worked with the NIHR Clinical Research Networks (CRNs) to contact hospitals across the UK asking for new expressions of interest (EOI). We were able to identify additional sites with the required clinical expertise, patient population and staff capacity.

#### Site set up and training

Site initiation training for pilot sites was delivered in person (pre-pandemic). Due to travel restrictions and to ensure safety of all staff, we redesigned and implemented remote site initiation training for phase II sites. This consists of an extensive package of training materials provided electronically, followed with a live online meeting for questions and answers, to discuss site-specific plans for study implementation and to identify any potential barriers. Remote training provides more flexibility for site staff to fit study training around their clinical commitments and their increased workload. We also developed a central electronic Investigator Site File (eISF) to replace paper files. This enables efficient provision of study documentation to sites teams, reduces the workload of site staff as it is managed by the central trial team, assists with ongoing maintenance and monitoring of the files, uses fewer physical resources and is more cost-effective.

#### Participant recruitment

Some NHS trusts introduced remote (telephone or video call) clinical appointments for their patients, where appropriate. Accordingly, we adapted study processes to enable patient identification and recruitment to proceed remotely, with no requirement for patients to be seen face-to-face at baseline. Site teams screen and identify potential participants (from referrals, clinic lists and during remote appointments) and provide study information to patients verbally, electronically or via post. We introduced electronic consent (eConsent), which was essential to facilitate the continuation of recruitment during the period when few face-to-face appointments and no interventions were being conducted. Site staff can randomise their participants and inform them of their allocated intervention via telephone. Through the eConsent system, participants complete their baseline questionnaire electronically during a remote appointment with recruiting staff (or verbally via telephone, if preferred).

#### Treatment (intervention)

The primary outcome for PURSUIT is the patient-reported ICIQ-UI-SF at 1 year after randomisation, but the huge increase in waiting list times for procedures means that patients may reach this critical study time point before they receive their treatment. To mitigate this risk, we introduced the option of delayed randomisation. Where waiting lists for interventions are around 12 months, site staff can proceed with taking full informed consent but do not randomise or collect baseline data until interventions can proceed.

#### Follow-up

There is no requirement for study-specific visits to hospital for follow-up. Participant questionnaires at all follow-up time points can be completed via post, electronically or over the telephone.

The adaptations described here do not replace the original in-person approach but are offered as alternatives to facilitate study delivery and will continue to be offered beyond the pandemic. Providing options to participants facilitates inclusivity which in turn supports recruitment and retention. Feedback regarding these changes from our patient and public involvement contributors was extremely positive and highlighted the importance of flexibility and individual preferences. Being able to participate remotely was considered to be of huge benefit to this particular patient group by reducing the burden associated with travel to hospital and reducing time away from home, work and childcare responsibilities.

The results from this study will provide the evidence which is currently lacking on endoscopic bulking injections and surgical treatments. The findings will inform future NICE policy and guidelines, providing healthcare professionals and patients with a solid evidence base to guide shared decision making regarding further treatment, ultimately improving symptoms and quality of life for women with recurrent SUI.

### Trial status

The current protocol is version 4.0 03SEP2020. Recruitment for the study started in January 2020 but we have experienced significant delays with recruitment due to the COVID-19 pandemic. We estimate recruitment to PURSUIT be completed around January 2024.

## Data Availability

The Data Manager (in collaboration with the CI) will manage access rights to the data set. Prospective new users must demonstrate compliance with legal, data protection and ethical guidelines before any data are released. Anonymous research data, including QRI audio-recordings and associated data, will be stored securely on research data storage facility (RDSF) and kept for future analysis with participant consent. We anticipate that anonymised trial data will be shared with other researchers to enable international prospective meta-analyses. Requests for access to data must be via a written confidentiality and DSA available from the RDSF website which will be confirmed by the CI (or appointed nominee). The DSA should cover limitations of use, transfer to third parties, data storage and acknowledgements. The person applying for use of the data will be scrutinised for appropriate eligibility by members of the research team.

## Abbreviations

AE: Adverse event
AUS: Artificial urinary sphincter
BTC: Bristol Trials Centre
BSUG: British Society of Urogynaecology
CI: Chief Investigator
CRF: Case report form
DMC: Data Monitoring Committee
DPA: Data Protection Act
EQ-5D-5L: EuroQol 5-Dimension 5-Level
GCP: Good Clinical Practice
GDPR: General Data Protection Regulation
HES: Hospital Episode Statistics
HCRW: Heath and Care Research Wales
HRA: Health Research Authority
HRG: Healthcare Resource Group
HTA: Health Technology Assessment
ICIQ-UI-SF: International Consultation on Incontinence Questionnaire Urinary Incontinence Short Form
eISF: Electronic Investigator Site File
ISRCTN: International Standard Randomised Controlled Trials Number
ITT: Intention to treat
MHRA: Medicines and Healthcare products Regulatory Agency
MUT: Midurethral Tape
NHS: National Health Service
NICE: National Institute for Health and Care Excellence
NIHR: National Institute for Health and Care Research
PI: Principal Investigator
PIL: Participant Information Leaflet
PISQ-IR: Pelvic Organ Prolapse/Urinary Incontinence Sexual Questionnaire-IUGA Revised
PGI-I: Patient Global Impression of Improvement
POP: Pelvic organ prolapse
PROM: Patient-reported outcome measure
QALY: Quality-adjusted life year
QoL: Quality of life
QRI: QuinteT Recruitment Intervention
QuinteT: Qualitative Research Integrated within Trials
RCT: Randomised control trial
REC: Research Ethics Committee
SAE: Serious adverse event
SAP: Statistical analysis plan
SD: Standard deviation
SOP: Standard Operating Procedure
SUI: Stress urinary incontinence
TMG: Trial Management Group
TOT: Transobturator Tape
TSC: Trial Steering Committee
TVT: Transvaginal Tape
UKCRC: United Kingdom Clinical Research Collaboration
UOB: University of Bristol
